# Maladaptive Denial of Severe Pain and Acute Orthopedic Injuries in a Patient With a Schizoaffective Disorder

**DOI:** 10.3389/fpsyg.2020.574673

**Published:** 2020-09-30

**Authors:** George P. Prigatano, Curtis McKnight, Megan Andrews, Jason Caplan

**Affiliations:** ^1^Department of Clinical Neuropsychology, Barrow Neurological Institute, St. Joseph’s Hospital and Medical Center, Phoenix, AZ, United States; ^2^Department of Psychiatry, Creighton School of Medicine, St. Joseph’s Hospital and Medical Center, Phoenix, AZ, United States; ^3^Department of Internal Medicine, School of Medicine, Creighton University, Omaha, NE, United States

**Keywords:** maladaptive denial, denial of illness questionnaire, severe pain, orthopedic injury, schizoaffective disorder

## Abstract

Persistent denial of severe and acute pain following orthopedic injuries has not been previously reported. We present a case of a 24-year-old woman with a history of schizoaffective disorder who suffered severe pain secondary to acute orthopedic injuries who insisted, “I am fine! There is nothing wrong with me.” Her maladaptive denial resulted in an initial refusal of necessary medical/surgical care, but she eventually accepted the necessary treatments despite her persistent belief she did not need such care. Her verbalizations and behaviors were characterized by active avoidance and angry reactions when a consulting psychiatrist spoke to her regarding her clinical condition. A modified version of the Conscious Avoidance subscale of the Denial of Illness Questionnaire was useful in measuring the severity level of her denial. This case report suggests that the behavioral features of psychological denial appear different from those associated impaired self-awareness secondary to an underlying brain disorder.

## Introduction

The scientific literature on disorders of consciousness and self-awareness is quite diverse and often focuses on either neurological explanations (Posner et al., 2007), different neuropsychological models (see Prigatano, 2010) or theories emanating from cognitive psychology (see Morin, 2006). To add to this complicated literature, different types and levels of conscious experiences have been described by several investigators which often use different terms when describing seemingly the same or similar phenomena (Morin, 2006). Depending on the patient group being studied, different models are favored in light of the scientific evidence. For example, there is little doubt that complete loss of conscious often involves damage or disruption of brain stem structures or is associated with diffuse cerebral dysfunction (Posner et al., 2007). Conversely, anosognosia for left hemiplegia is often associated with unequivocal damage to the right parietal-frontal circuitry which also often directly or indirectly effects the insular cortex (Vocat and Vuilleumier, 2010). Understanding persistent impaired self-awareness (ISA) in patients with post-acute severe traumatic brain injury (TBI) has been more challenging. Recently Prigatano and Sherer (2020) noted that research on the neurological correlates of commonly used measures of ISA in post-acute TBI have revealed only moderate size effects. They suggest this may due to the fact denial phenomena contributes to some TBI patients underreporting their neuropsychological and behavioral limitations. The potential role of denial in the clinical presentation of diverse patient groups has been recognized for several years (Weinstein and Kahn, 1955).

Denial has repetitively been described by psychiatric clinicians as an automatic, non-conscious psychological method of coping with intensely anxiety producing situations (American Psychiatric Association, 1994). Denial can be observed in various medical and psychiatric conditions, including alcohol addiction (Paredes, 1974), psychoses (Amador and David, 2004), cancer (Pene and Kissane, 2019), and heart disease (Livneh, 2009), including acute myocardial infarction (Olin and Hackett, 1964). While denial may serve to consciously reduce the perception of a threat that aids momentary adaptation (Lazarus, 1983), severe and persistent denial can have quite adverse consequences for individuals as they fail to obtain necessary psychological and medical care.

One interesting form of denial centers on the denial of pain. Most conceptual models of consciousness recognize the existence of “sensorimotor awareness” which includes the perception of pain (Morin, 2006). Conscious awareness of pain is thought to be mediated by deep brain structures involving the brainstem and thalamus (Stuss and Anderson, 2004). Yet, denying the existence of pain can readily be observed in patients within intact brainstem and subcortical functions. This suggests that non-neurological factors can influence the conscious perception (i.e., subjective experience) of pain.

Weinstein and Kahn described a phenomenon they referred to as “pain asymbolia.” They noted that some patients deny experiencing pain when pricked with a needle. However, we could find no reports of denial of severe pain that has an acute onset. Severe acute pain, by its nature, is such an aversive experience that the individual is often prone to quickly acknowledge it and seek immediate efforts at relief. It is unusual, therefore, to be confronted with a patient who has the onset of acute severe pain and denies the pain. Even patients who are experiencing chest pain secondary to acute myocardial infarction do not deny the pain *per se*, but they often attribute it to benign causes (Olin and Hackett, 1964).

The description and associated measurement of denial of acute onset of severe pain may provide clues as to how denial phenomena can be measured and ultimately be separated from measures of ISA that appear to be a direct result of underlying brain dysfunction. In this brief case report, we describe a young woman who was obviously in severe pain following acute orthopedic injuries who denied her pain and for a period of time refused necessary medical and surgical treatments. We briefly describe her clinical course, behavioral characteristics, and psychosocial history that are relevant for understanding such dramatic behaviors. Her psychiatric management is also briefly summarized. Following the suggestion of Prigatano and Sherer (2020) of the potential use of the Conscious Avoidance subscale of the Illness Denial Questionnaire (IDQ) for measuring clinically significant levels of denial, we specifically evaluated this patient’s behavior and verbalizations using this subscale. We conclude with suggestions for briefly assessing medical and psychiatric patients suspected of presenting with clinically significant (maladaptive) denial.

## Methods

### Case Report

Ms. M, a 24-year-old woman with history of schizoaffective disorder, was emergently brought to a Level 1 Trauma Center after being struck by a car while riding her bicycle. Examination revealed a T7 burst fracture, left intra-articular distal humerus fracture, and retroperitoneal hematoma. According to the first responders and witnesses, she did not lose consciousness, and a head CT scan was unremarkable. In the trauma bay, Ms. M denied that she had suffered any injuries, refused care, and asked to leave against medical advice (AMA). Psychiatry was consulted to assess her capacity to do so.

At the time of the initial psychiatric assessment, Ms. M had been moved to the intensive care unit. She was awake, in physical distress, diaphoretic and tachycardic. After introductions, she responded, “There’s nothing wrong with me. The only problem is I have to use the bathroom. Help me up.” She interrupted attempts to discuss and explain her injuries and went from initially impatient to openly hostile as discussions continued. Further attempts to discuss and review her injuries led to her shouting for the consultant to leave the room. She dismissed the obvious deformity of her left arm and her facial abrasions and contusions, simply reiterating, “No, I’m fine!” She was found incapacitated to leave AMA, and her father was identified as her surrogate decision maker. He authorized her further medical care.

Over the next 5 days of her hospital stay, her anger related to discussion of injuries slowly diminished. On hospital day 4 she indicated, “They say I was hit by a car, but I don’t know.” She was less hostile and more willing to engage in a psychiatric assessment. Still, she continued to change the topic or became guarded when her injuries were discussed. She acknowledged that she had been diagnosed with schizoaffective disorder in the past and was agreeable to taking Quetiapine. By hospital day 6, she was superficially agreeable to surgery, noting, “My doctors say I need it, so I’m okay with it.” She underwent the necessary surgeries for her humerus and spinal fractures with the informed consent of her father. By hospital day 15, she was cooperative but had inappropriately bright affect. She participated in physical and occupational therapy but often commented, “This isn’t worth the trouble” and “Deep down I don’t think there’s anything wrong with me.” She did none of the assigned homework exercises from physical therapy and occupational therapy but did participate in sessions with substantial coaxing. She was transferred to a dedicated inpatient psychiatric facility on hospital day 35.

During her hospital admission, her father provided the following history regarding his daughter. Ms. M was born to an American man and a Peruvian woman. She was raised in the United States, but frequently visited Peru growing up. In her late teenage years after high school, she moved to Peru, married, and had a daughter. About a year after the birth of her daughter, she developed mania with psychosis and was psychiatrically hospitalized in Peru for several months. She was eventually stabilized on Lithium and cleared to travel. She returned to the United States and in a few months self-discontinued her medications and decompensated. Her father noted that throughout her life and prior to her present injuries this patient always seemed to avoid discussing emotionally charged topics.

### Measurement of Denial

There presently does not exist a psychometrically validated scale for measuring denial of pain. However, the IDQ for patients and caregivers (Ferrario et al., 2017) might be adapted for this purpose. As noted above, Prigatano and Sherer (2020) suggested that the Conscious Avoidance subscale of the IDQ may be useful in assessing some aspects of denial phenomena. After the patient was evaluated and treated, as described in the case report, her behavior and verbalizations were retrospectively rated by the first and second author using the Conscious Avoidance subscale. This scale has eight behavioral items/questions which characterize avoidance behaviors associated with denial of physical illness. They are reproduced (with permission) in Table 1.

**TABLE 1:** Conscious avoidance subscale of the illness denial questionnaire (IDQ) from Ferrario et al. (2017).

**IDQ Subscale Items^a^**
Item 1. I try to avoid thinking about this disorder/disease as much as I can.
Item 2. I try not to pay attention to my disorder/disease.
Item 3. I try not to speak about this disorder/disease with the doctor or other specialists.
Item 4. I do not want to have to look the disorder/disease in the face.
Item 5. The less I know, the better I feel.
Item 6. I try not to speak about this disorder/disease.
Item 7. At times I try to convince myself that I do not have any disorder/disease.
Item 8. The best way to cope with this disorder/disease is to not think about it.
Additional Suggested Items
Item 9. The patient actively discourages the clinician from further inquiry regarding his or her clinical condition.
Item 10. The patient becomes hostile and/or openly angry with further questions are raised by the clinician.

*^*a*^Ferrario et al. (2017) Reprinted with permission.*

Given the clinical findings described in the Case Report, two additional items were suggested as further indications of denial not included in the Conscious Avoidance subscale (see Table 1). They were not psychometrically validated, but are included for potential heuristic value.

## Results and Discussion

Patients diagnosed with a schizoaffective disorder are notoriously difficult to treat because of their impaired insight into their psychiatric disturbance (Amador and David, 2004). They not only are resistant to taking medications, but it can be difficult to establish a therapeutic relationship with them. Without proper psychotropic medications and psychotherapy, their psychiatric status can deteriorate with the emergence of more severe psychotic behaviors. This appeared to have occurred in this patient. However, her denial of severe acute pain is highly unusual despite her psychiatric diagnosis. We could find no reports in the literature that describe denial of severe pain with associated acute orthopedic injuries in a patient with or without a psychotic disorder. Moreover, the patient described in this case report acknowledged her psychiatric diagnosis and was agreeable to taking psychotropic medication. Her maladaptive denial was specifically about having suffered acute injuries and being in severe pain.

The findings of this case report are difficult to explain if one limits one’s understanding of disturbed conscious awareness to neurological, neuropsychology or cognitive psychology models. There was no evidence of neurological injuries especially to brainstem or thalamus. There was no evidence of underlying neuropsychological difficulties that could account for this patient’s intense verbal responses and behaviors regarding her pain. Her disturbed “sensorimotor awareness” was characterized by emotional reactions not considered in cognitive models of conscious awareness (Morin, 2006) nor observed in patients with underlying brain pathology (Prigatano, 2010). Rather this patient’s clinical presentation is best explained on the basis of psychodynamic models that emphasize denial is associated with avoidance of anxiety producing experiences.

Anxiety can be caused by a number of factors, but a long standing series of studies in psychiatry have shown a connection between attachment styles developed early in life and different types of anxiety patterns observed in adulthood (Bowlby and King, 2004). Poor insight has been specifically related to insecure attachment styles in patients with schizophrenia (Franca et al., 2019). Korver-Nieberg et al. (2015) also noted that avoidant and anxiety attachment histories are frequently associated with psychotic behaviors, including severe (psychotic appearing) denial. This patient’s psychosocial history is certainly compatible with significant attachment difficulties during childhood that extended into her adult life. Such individuals often do not trust others who might be broadly described as surrogate parental figures (e.g., doctors). Korver-Nieberg et al. (2015) note that regular (often daily) predictable psychotherapeutic sessions that acknowledge the patient’s experiences via empathetic listening can help build a therapeutic alliance which results in a reduction in psychotic behaviors. In the course of managing this patient, a similar approach was used and eventually she agreed to treatment even though she consciously continued to doubt that such treatment was necessary.

Denial is often conceptualized as an “automatic psychological process that protects the individual against anxiety and from awareness of internal or external stressors” (American Psychiatric Association, 1994, pg. 765). While clinically significant, maladaptive denial can be associated with various cognitive, emotional, and behavioral reactions (Prigatano and Sherer, 2020), one defining feature has been the phenomenon of actively avoiding a discussion of what problems the person attempts to deny. The patient may not respond to questions or may attempt to change the topic of discussion. The individual may indicate a wish not to talk about or think about a problem he or she is facing. When pressed to talk about her clinical condition, the patient can become progressively angry and hostile. This was the pattern observed in the patient described in this brief clinical report. In light of these observations and recent suggestions regarding measuring denial and separating it from anosognosia (Prigatano and Sherer, 2020), we rated our patient’s behavior on the Conscious Avoidance subscale of the IDQ (12). We also added two additional items to capture the clinical phenomena of anger associated with discussing topics the patient wished to avoid.

### The Patient’s Verbalizations and Behavior as Rated on the Conscious Avoidance Subscale of the Illness Denial Questionnaire

Eight items compose the Conscious Avoidance subscale of the IDQ. They are listed in Table 1. Given the findings summarized in this case report, avoidance behaviors appeared to dominate the major features of this patient’s denial of acute and severe pain. This patient demonstrated seven of the eight behavioral characteristics suggesting active conscious avoidance of her clinical condition. She tried to avoid thinking and talking about her disorder/clinical condition (Items 1, 3, and 6). She was convinced that there was nothing wrong with her (Item 7). She felt that the doctors should leave her alone and not “look at” or “think about” her clinical condition (Items 2, 4, and 8). The only item (Item 5) that did not reflect her behavior was the statement: “The less I know, the better I feel.” This was not explicitly stated by the patient, but the statement “I am fine, leave me alone” suggests it.

While useful, the Conscious Avoidance subscale does not include items that specifically reflect anger or hostile reactions. The clinical presentation of this patient also highlights angry and hostile reactions. These dimensions are not included in the Conscious Avoidance subscale and consequently we suggest adding two additional items when quantifying clinically severe (or maladaptive) denial. They are: (1) the patient demonstrates behaviors that actively discourage the clinician from further inquiries about the patient’s clinical condition (Item 9) and (2) the patient becomes hostile or openly angry (often yelling) at the clinician’s further attempts to explore the patient’s clinical condition despite the obvious efforts at discouraging such inquiries (Item 10). Using these 10 items, we found the patient demonstrated nine out of the ten behavioral features suggestive of clinically significant (maladaptive) denial. Future group studies will be needed, of course, to determine whether the Conscious Avoidance subscale of the IDQ with the additional two items is useful in measuring denial in different patient groups as suggested by Prigatano and Sherer (2020).

### Limitations and Future Research

There are numerous limitations to the present case report that need to be acknowledged. The patient was seen for a short period of time at the request of her physicians for inpatient psychiatric consultation. We do provide details regarding her medical/surgical management/treatment since she was seen for a relatively brief period of time. The major focus of this case report was not on her psychiatric treatment *per se*, but the characterization and brief measurement of her denial of acute and severe orthopedic pain. We do not have extended follow-up information regarding her clinical condition as indicated in this report. We did not use standardized measures to arrive at her psychiatric diagnosis. It was based purely on her psychiatric history. We did not employee standardized measures of anxiety or depression. We did not employee standardized neuropsychological tests to describe her cognitive functioning. However, clinically the patient appeared to understand what was being communicated to her and there was no evidence of acquired brain dysfunction associated with her injures.

Future research in the area of denial of pain as well as the denial of other obvious disabilities or impairments (such as severe memory impairment or inability to walk associated with spinal cord injuries) may benefit from utilizing the Conscious Avoidance subscale of the IDQ. The present report is limited to a case study and future group studies are needed to evaluate the clinical and research utility of the Conscious Avoidance subscale of the IDQ. Future research should also explore the patient’s emotional reactions when their area of denial is directly addressed by the clinician. These reactions may help separate denial as a psychological method of coping from ISA as a neuropsychological impairment as originally suggested by Prigatano and Klonoff (1998) and more recently by Prigatano and Sherer (2020).

## Summary and Conclusion

We presented a case of a 24-year-old woman with schizoaffective disorder who denied suffering severe pain associated with acute orthopedic injuries. This unusual case highlights important issues in the early psychiatric management of such patients and emphasizes that maladaptive denial is often associated with both avoidance behaviors and angry reactions when the object of their denial is openly spoken about with them. A modified version the Conscious Avoidance subscale of the IDQ may be a useful measure for rating severity of maladaptive denial.

## Ethics Statement

This case report involving a human subject was reviewed and approved by the Corporate Responsibility Office of Common Spirit Health (Parent Company of St. Joseph’s Hospital and Medical Center, Phoenix, Arizona and the Barrow Neurological Institute, Phoenix, Arizona). This office waived the requirement for consent. Written informed consent from the patient and legal guardian was not required to participate in this study in accordance with the national legislation and the institutional requirements.

## Author Contributions

GP: literature review, conceptualization of the case study, and discussion. CM, MA, and JC: writing of case study. All authors contributed to the article and approved the submitted version.

## Conflict of Interest

The authors declare that the research was conducted in the absence of any commercial or financial relationships that could be construed as a potential conflict of interest.
